# A Bioconductor workflow for the Bayesian analysis of spatial proteomics

**DOI:** 10.12688/f1000research.18636.1

**Published:** 2019-04-11

**Authors:** Oliver M. Crook, Lisa M. Breckels, Kathryn S. Lilley, Paul D.W. Kirk, Laurent Gatto

**Affiliations:** 1 Cambridge Centre for Proteomics, Department of Biochemistry, University of Cambridge, Cambridge, CB2 1QR, UK; 2 MRC Biostatistics Unit, Cambridge Institute for Public Health, University of Cambridge, Cambridge, CB2 0SR, UK; 3 Université catholique de Louvain, Brussels, 1200, Belgium

**Keywords:** pRoloc, pRolocdata, proteomics, Bayesian, software, Bioconductor, spatial proteomics, machine learning

## Abstract

Knowledge of the subcellular location of a protein gives valuable insight into its function. The field of spatial proteomics has become increasingly popular due to improved multiplexing capabilities in high-throughput mass spectrometry, which have made it possible to systematically localise thousands of proteins per experiment. In parallel with these experimental advances, improved methods for analysing spatial proteomics data have also been developed. In this workflow, we demonstrate using `pRoloc` for the Bayesian analysis of spatial proteomics data. We detail the software infrastructure and then provide step-by-step guidance of the analysis, including setting up a pipeline, assessing convergence, and interpreting downstream results. In several places we provide additional details on Bayesian analysis to provide users with a holistic view of Bayesian analysis for spatial proteomics data.

## Introduction

Determining the the spatial subcellular distribution of proteins enables novel insight into protein function ^1^. Many proteins function within a single location within the cell; however, it is estimated that up to half of the proteome is thought to reside in multiple locations, with some of these undergoing dynamic relocalisation ^2^. These phenomena lead to variability and uncertainty in robustly assigning proteins to a unique localisation. Functional compartmentalisation of proteins allows the cell to control biomolecular pathways and biochemical processes within the cell. Therefore, proteins with multiple localisations may have multiple functional roles ^3^. Machine learning algorithms that fail to quantify uncertainty are unable to draw deeper insight into understanding cell biology from mass spectrometry (MS)-based spatial proteomics experiments. Hence, quantifying uncertainty allows us to make rigorous assessments of protein subcellular localisation and multi-localisation.

For proteins to carry out their functional role they must be localised to the correct subcellular compartment, ensuring the biochemical conditions for desired molecular interactions are met ^4^. Many pathologies, including cancer and obesity are characterised by protein mis-localisations ^5– 14^. High-throughput spatial proteomics technologies have seen rapid improvement over the last decade and now a single experiment can provide spatial information on thousands of proteins at once ^15– 18^. As a result of these spatial proteomics technologies many biological systems have been characterised ^2, 15, 17, 19– 21^. The popularity of such methods is now evident with many new studies in recent years ^17, 22– 29^.

Bayesian approaches to machine learning and statistics can provide more insight, by providing uncertainty quantification ^30^. In a parametric Bayesian setting, a parametric model is proposed, along with a statement about our prior beliefs of the model parameters. Bayes’ theorem tells us how to update the prior distribution of the parameters to obtain the posterior distribution of the parameters after observing the data. It is the posterior distribution which quantifies the uncertainty in the parameters. This contrasts from a maximum-likelihood approach where we obtain only a point estimate of the parameters.

Adopting a Bayesian framework for data analysis, though of much interest to experimentalists, can be challenging. Once we have specified a probabilistic model, computational approaches are typically used to obtain the posterior distribution upon observation of the data. These algorithms can have parameters that require tuning and a variety of settings, hindering their practical use by those not familiar with Bayesian methodology. Even once the algorithms have been correctly set-up, assessments of convergence and guidance on how to interpret the results are often sparse. This workflow presents a Bayesian analysis of spatial proteomics to elucidate the process for practitioners. Our workflow also provides a template for others interested in designing tools for the biological community which rely on Bayesian inference.

Our model for the data is the t-augmented Gaussian mixture (TAGM) model proposed in 1. Crook *et al*. ^1^ provide a detailed description of the model, rigorous comparisons and testing on many spatial proteomics datasets, including a case study in which a hyperLOPIT experiment is performed on mouse pluripotent stem cells ^17, 31^. Revisiting these details is not the purpose of this computational protocol; rather we present how to correctly use the software and provide step-by-step guidance for interpreting the results.

In brief, the TAGM model posits that each annotated sub-cellular niche can be modelled using a Gaussian distribution. Thus the full complement of proteins within the cell is captured as a mixture of Gaussians. The highly dynamic nature of the cell means that many proteins are not well captured by any of these multivariate Gaussian distributions, and thus the model also includes an outlier component, which is mathematically described as a multivariate student’s t distribution. The heavy tails of the t distribution allow it to better capture dispersed proteins.

There are two approaches to perform inference in the TAGM model. The first, which we refer to as TAGM MAP, allows us to obtain *maximum a posteriori* estimates of posterior localisation probabilities; that is, the modal posterior probability that a protein localises to that class. This approach uses the expectation-maximisation (EM) algorithm to perform inference ^32^. Whilst this is a interpretable summary of the TAGM model, it only provides point estimates. For a richer analysis, we also present a Markov-chain Monte-Carlo (MCMC) method to perform fully Bayesian inference in our model, allowing us to obtain full posterior localisation distributions. This method is referred to as TAGM MCMC throughout the text.

This workflow begins with a brief review of some of the basic features of mass spectrometry-based spatial proteomics data, including our state-of-the-art computational infrastructure and bespoke software suite. We then present each method in turn, detailing how to obtain high quality results. We provide an extended discussion of the TAGM MCMC method to highlight some of the challenges that may arise when applying this method. This includes how to assess convergence of MCMC methods, as well as methods for manipulating the output. We then take the processed output and explain how to interpret the results, as well as providing some tools for visualisation. We conclude with some remarks and directions for the future. Source code for this workflow, including code used to generate tables and figures, is available on GitHub ^33^

## Getting started and infrastructure

In this workflow, we are using version 1.23.2 of pRoloc ^34^. The package pRoloc contains algorithms and methods for analysing spatial proteomics data, building on the MSnSet structure provided in MSnbase. The pRolocdata package provides many annotated datasets from a variety of species and experimental procedures. The following code chunks install and load the suite of packages require for the analysis.

if (!require("BiocManager"))
    install.package("BiocManager")
BiocManager::install(c("pRoloc", "pRolocdata"))

library("pRoloc")

##
## This is pRoloc version 1.23.2
##   Visit https://lgatto.github.io/pRoloc/ to get started.

library("pRolocdata")

##
## This is pRolocdata version 1.21.1.
## Use ’pRolocdata()’ to list available data sets.

We assume that we have a MS-based spatial proteomics dataset contained in a MSnSet structure. For information on how to import data, perform basic data processing, quality control, supervised machine learning and transfer learning we refer the reader to 35. Here, we start by loading a spatial proteomics dataset on mouse E14TG2a embryonic stem cells ^36^. The LOPIT protocol ^15, 37^ was used and the normalised intensity of proteins from eight iTRAQ 8-plex labelled fraction are provided. The methods provided here are independent of labelling procedure, fractionation process or workflow. Examples of valid experimental protocols are LOPIT ^37^, hyperLOPIT ^17, 31^, label-free methods such as PCP ^16^, and when fractionation is perform by differential centrifugation ^18, 38^.

In the code chunk below, we load the aforementioned dataset. The printout demonstrates that this experiment quantified 2031 proteins over 8 fractions.

data("E14TG2aR") # load experimental data
E14TG2aR

## MSnSet (storageMode: lockedEnvironment)
## assayData: 2031 features, 8 samples
##   element names: exprs
## protocolData: none
## phenoData
##   sampleNames: n113 n114 ... n121 (8 total)
##   varLabels: Fraction.information
##   varMetadata: labelDescription
## featureData
##   featureNames: Q62261 Q9JHU4 ... Q9EQ93 (2031 total)
##   fvarLabels: Uniprot.ID UniprotName ... markers (8 total)
##   fvarMetadata: labelDescription
## experimentData: use ’experimentData(object)’
## Annotation:
## - - - Processing information - - -
## Loaded on Thu Jul 16 15:02:29 2015.
## Normalised to sum of intensities.
## Added markers from ’mrk’ marker vector. Thu Jul 16 15:02:29 2015
##  MSnbase version: 1.17.12

**Figure 1.**
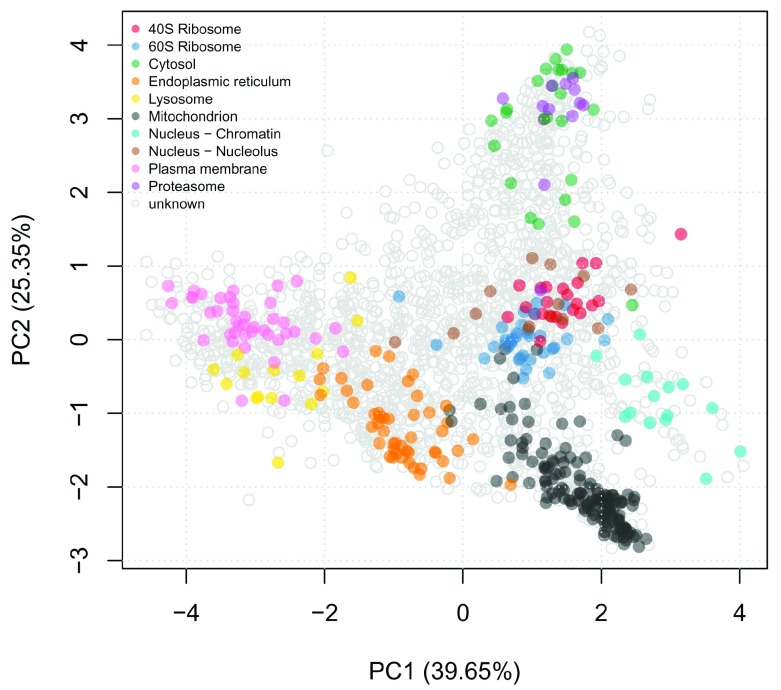
First two principal components of mouse stem cell data.

In Figure 1, we can visualise the mouse stem cell dataset use the plot2D function. We observe that some of the organelle classes overlap and this is a typical feature of biological datasets. Thus, it is vital to perform uncertainty quantification when analysing biological data.

plot2D(E14TG2aR)
addLegend(E14TG2aR, where = "topleft", cex = 0.6)

## Methods: TAGM MAP

### Introduction to TAGM MAP

We can use *maximum a posteriori* (MAP) estimation to perform Bayesian parameter estimation for our model. The *maximum a posteriori* estimate is the mode of the posterior distribution and can be used to provide a point estimate summary of the posterior localisation probabilities. In contrast to TAGM MCMC (see later), it does not provide samples from the posterior distribution, however it allows for faster inference by using an extended version of the expectation-maximisation (EM) algorithm. The EM algorithm iterates between an expectation step and a maximisation step. This allows us to find parameters which maximise the logarithm of the posterior, in the presence of latent (unobserved) variables. The EM algorithm is guaranteed to converge to a local mode. The code chunk below executes the tagmMapTrain function for a default of 100 iterations. We use the default priors for simplicity and convenience, however they can be changed, which we explain in a later section. The output is an object of class MAPParams, that captures the details of the TAGM MAP model.

set.seed(2)
mappars <- tagmMapTrain(E14TG2aR)

## co-linearity detected; a small multiple of
##               the identity was added to the covariance

mappars

## Object of class "MAPParams"
##  Method: MAP

### Aside: collinearity

The previous code chunk outputs a message concerning data collinearity. This is because the covariance matrix of the data has become ill-conditioned and as a result the inversion of this matrix becomes unstable with floating point arithmetic. This can lead to the failure of standard matrix algorithms upon which our method depends. In this case, it is standard practice to add a small multiple of the identity to stabilise this matrix. The printed message is a statement that this operation has been performed for these data.

### Model visualisation

**Figure 2.**
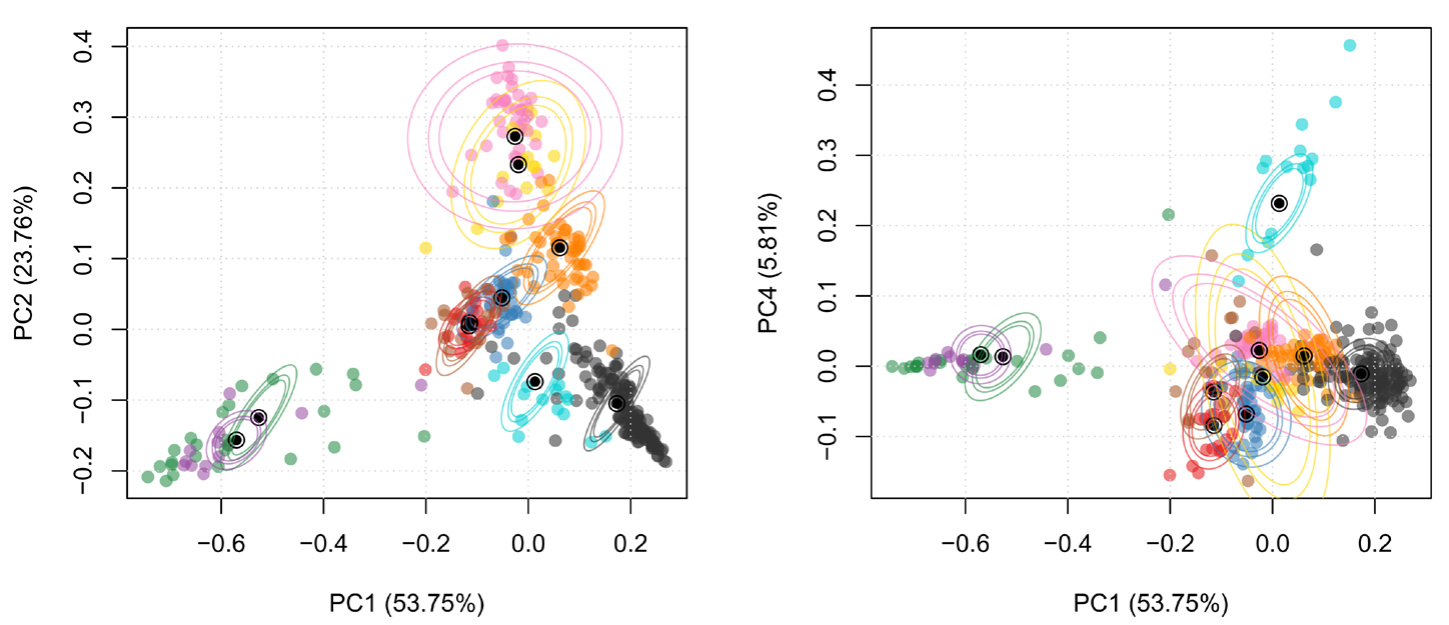
PCA plot with probability ellipses along PC 1 and 2 (left) and PC 1 and 4 (right).

The results of the modelling can be visualised with the plotEllipse function on Figure 2. The outer ellipse contains 99% of the total probability whilst the middle and inner ellipses contain 95% and 90% of the probability respectively. The centres of the clusters are represented by black circumpunct (circled dot). We can also plot the model in other principal components. The code chunk below plots the probability ellipses along the first and second, as well as the fourth principal component. The user can change the components visualised by altering the dims argument.

par(mfrow = c(1, 2))
plotEllipse(E14TG2aR, mappars)
plotEllipse(E14TG2aR, mappars, dims = c (1, 4))

### The expectation-maximisation algorithm

**Figure 3.**
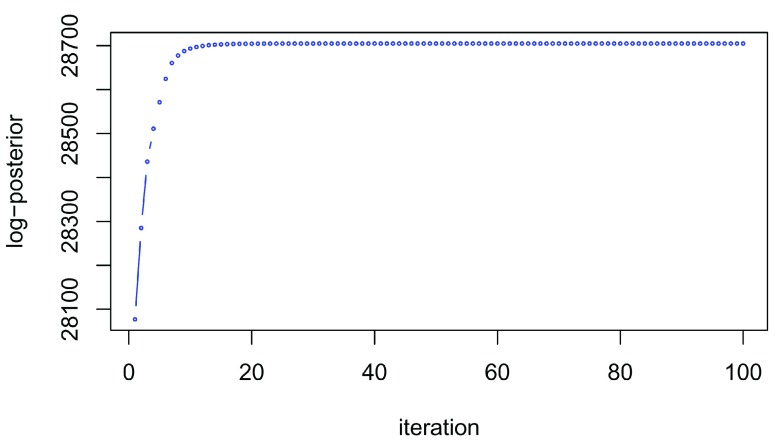
Log-posterior at each iteration of the EM algorithm demonstrating convergence.

The EM algorithm is iterative; that is, the algorithm iterates between an expectation step and a maximisation step until the value of the log-posterior does not change ^32^. This fact can be used to assess the convergence of the EM algorithm. The value of the log-posterior at each iteration can be accessed with the logPosteriors function on the MAPParams object. The code chuck below plots the log posterior at each iteration and we see on Figure 3 the algorithm rapidly plateaus and so we have achieved convergence. If convergence has not been reached during this time, we suggest to increase the number of iterations by changing the parameter numIter in the tagmMapTrain method. In practice, it is not unexpected to observe small fluctuations due to numerical errors and this should not concern users.

plot(logPosteriors(mappars), type = "b", col = "blue",
      cex = 0.3, ylab = "log-posterior", xlab = "iteration")

The code chuck below uses the mappars object generated above, along with the E14RG2aR dataset, to classify the proteins of unknown localisation using tagmPredict function. The results of running tagmPredict are appended to the fData columns of the MSnSet.

E14TG2aR <- tagmPredict(E14TG2aR, mappars) # Predict protein localisation

The new feature variables that are generated are:

- tagm.map.allocation: the TAGM MAP predictions for the most probable protein sub-cellular allocation.

table(fData(E14TG2aR)$tagm.map.allocation)

##
##          40S Ribosome          60S Ribosome               Cytosol
##                    34                    85                   328
## Endoplasmic reticulum              Lysosome         Mitochondrion
##                   284                   147                   341
##   Nucleus - Chromatin   Nucleus - Nucleolus       Plasma membrane
##                   143                   322                   326
##            Proteasome
##                    21

- tagm.map.probability: the posterior probability for the protein sub-cellular allocations.

summary(fData(E14TG2aR)$tagm.map.probability)

##     Min. 1st Qu.  Median    Mean 3rd Qu.    Max.
##  0.00000 0.06963 0.93943 0.63829 0.99934 1.00000

- tagm.map.outlier: the posterior probability for that protein to belong to the outlier component rather than any annotated component.

summary(fData(E14TG2aR)$tagm.map.outlier)


##      Min.   1st Qu.    Median      Mean   3rd Qu.      Max.
## 0.0000000 0.0002363 0.0305487 0.3452624 0.9249810 1.0000000

**Figure 4.**
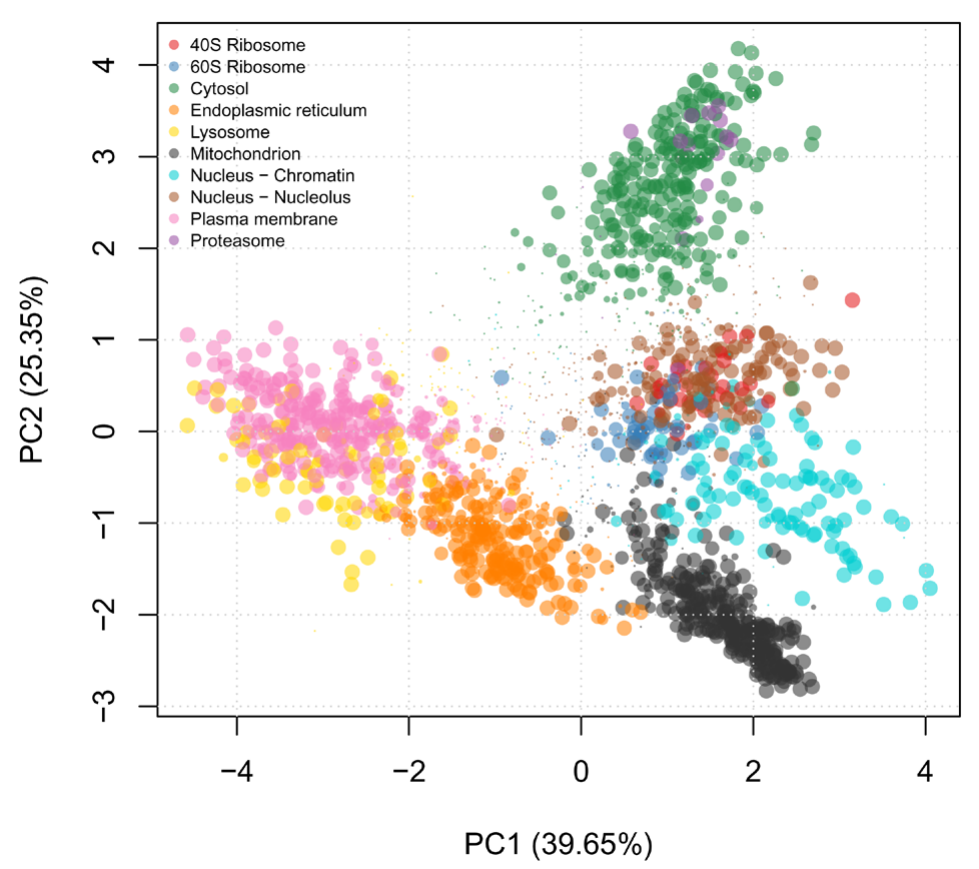
TAGM MAP allocations, where the pointer is scaled according to the localisation probability and coloured according to the most probable subcellular niche.

We can visualise the results by scaling the pointer according the posterior localisation probabilities. To do this we extract the MAP localisation probabilities from the feature columns of the the MSnSet and pass these to the plot2D function ( Figure 4).

ptsze <- fData(E14TG2aR)$tagm.map.probability # Scale pointer size
plot2D(E14TG2aR, fcol = "tagm.map.allocation", cex = ptsze)
addLegend(E14TG2aR, where = "topleft", cex = 0.6, fcol = "tagm.map.allocation")

The TAGM MAP method is easy to use and it is simple to check convergence, however it is limited in that it can only provide point estimates of the posterior localisation distributions. To obtain the full posterior distributions and therefore a rich analysis of the data, we use Markov-Chain Monte-Carlo methods. In our particular case, we use a *collapsed Gibbs sampler* ^39^.

## Methods: *TAGM MCMC* a brief overview

The TAGM MCMC method allows a fully Bayesian analysis of spatial proteomics datasets. It employs a collapsed Gibbs sampler to sample from the posterior distribution of localisation probablities, providing a rich analysis of the data. This section demonstrates the advantage of taking a Bayesian approach and the biological information that can be extracted from this analysis.

For those unfamiliar with Bayesian methodology, some of the key ideas for a more complete understanding are as follows. Firstly, MCMC based inference contrasts with MAP based inference in that it *samples* from the posterior distribution of localisation probabilities. Hence, we do not just have a single estimate for each quantity but a distribution of estimates. MCMC methods are a large class of algorithms used to sample from a probability distribution, in our case the posterior distribution of the parameters ^40^. Once we have sampled from the posterior distribution, we can estimate the mean of the posterior distribution by simply taking the mean of the samples. In a similar fashion, we can obtain estimates of other summaries of the posterior distribution.

**Figure 5.**
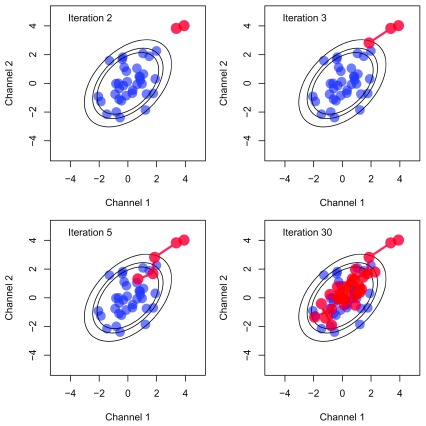
A schematic figure of MCMC sampling. Proteins are coloured in blue and probability ellipses are overlaid representing contours of a probability distribution matching the distribution of the proteins. MCMC samples from the mean of this distribution are then coloured in red.

A schematic of MCMC sampling is provided in Figure 5 to aid understanding. Proteins, coloured blue, are visualised along two variables of the data. Probability ellipses representing contours of a probability distribution matching the distribution of the proteins are overlaid. We now wish to obtain samples from this distribution. The MCMC algorithm is initialised with a starting location, then at each iteration a new value is proposed. These proposed values are either accepted or rejected (according to a carefully computed acceptance probability) and over many iterations the algorithm converges and produces samples from the desired distribution. Samples from the mean of this distribution are coloured in red in the schematic figure. A large portion of the earlier samples may not reflect the true distribution, because the MCMC sampler has yet to converge. These early samples are usually discarded and this is referred to as burn-in. The next state of the algorithm depends on its current state and this leads to auto-correlation in the samples. To suppress this auto-correlation, we only retain every *r ^th^* sample. This is known as thinning. The details of burn-in and thinning are further explained in later sections.

The TAGM MCMC method is computationally intensive and requires at least modest processing power. Leaving the MCMC algorithm to run overnight on a modern desktop is usually sufficient, however this, of course, depends on the particular dataset being analysed. For guidance: it should not be expected that the analysis will finish in just a couple of hours on a medium specification laptop, for example.

To demonstrate the class structure and expected outputs of the TAGM MCMC method, we run a brief analysis on a subset (400 randomly chosen proteins) of the tan2009r1 dataset from the pRolocdata, purely for illustration. This is to provide a bare bones analysis of these data without being held back by computational requirements. We perform a complete demonstration and provide precise details of the analysis of the stem cell dataset considered above in the next section.

set.seed(1)
data(tan2009r1)
tan2009r1 <- tan2009r1[sample(nrow(tan2009r1),400), ]


The first step is to run a few MCMC chains (below we use only 2 chains) for a few iterations (we specify 3 iterations in the below code, but typically we would suggest in the order of tens of thousands; see for example the algorithms default settings by typing ?tagmMcmcTrain) using the tagmMcmcTrain function. This function will generate a object of class MCMCParams.

p <- tagmMcmcTrain(object = tan2009r1, numIter = 3,
                     burnin = 1, thin = 1, numChains = 2)
p


## Object of class "MCMCParams"
## Method: TAGM.MCMC
## Number of chains: 2

Information for each MCMC chain is contained within the chains slot. If needed, this information can be accessed manually. The function tagmMcmcProcess processes the MCMCParams object and populates the summary slot.

p <- tagmMcmcProcess(p)
p


## Object of class "MCMCParams"
## Method: TAGM.MCMC
## Number of chains: 2
## Summary available



The summary slot has now been populated to include basic summaries of the MCMC chains, such as organelle allocations and localisation probabilities. Protein information can be appended to the feature columns of the MSnSet by using the tagmPredict function, which extracts the required information from the summary slot of the MCMCParams object.

res <- tagmPredict(object = tan2009r1, params = p)


We can now access new variables:

- tagm.mcmc.allocation: the TAGM MCMC prediction for the most likely protein sub-cellular annotation.

table(fData(res)$tagm.mcmc.allocation)

##
##  Cytoskeleton              ER        Golgi      Lysosome   mitochondrion
##            11              98           22             9              40
##       Nucleus      Peroxisome           PM    Proteasome    Ribosome 40S
##            25               3          104            29              31
##  Ribosome 60S
##            28

- tagm.mcmc.probability: the mean posterior probability for the protein sub-cellular allocations.

summary(fData(res)$tagm.mcmc.probability)



##   Min.  1st Qu.  Median    Mean  3rd Qu.   Max.
## 0.3035   0.8974  0.9889  0.9088   1.0000 1.0000



We can also access other useful summaries of the MCMC methods:

- tagm.mcmc.outlier the posterior probability for the protein to belong to the outlier component.
- tagm.mcmc.probability.lowerquantile and tagm.mcmc.probability.upperquantile are the lower and upper boundaries to the equi-tailed 95% credible interval of tagm.mcmc.probability.
- tagm.mcmc.mean.shannon a Monte-Carlo averaged Shannon entropy, which is a measure of uncertainty in the allocations.

## Methods: *TAGM MCMC* the details

This section explains how to manually manipulate the MCMC output of the TAGM model. In the code chunk below, we load a pre-computed TAGM MCMC model. The data file e14tagm.rda is available online ^1^ and is not directly loaded into this package due to its size. The file itself if around 500mb, which is too large to directly load into a package.

load("e14Tagm.rda")

The following code, which is not evaluated dynamically, was used to produce the tagmE14 MCMCParams object. We run the MCMC algorithm for 20,000 iterations with 10,000 iterations discarded for burn-in. We then thin the chain by 20. We ran 6 chains in parallel and so we obtain 500 samples for each of the 6 chains, totalling 3,000 samples. The resulting file is assumed to be in our working directory.

e14Tagm <- tagmMcmcTrain(E14TG2aR,
                            numIter = 20000,
                            burnin = 10000,
                            thin = 20,
                            numChains = 6)

Manually inspecting the object, we see that it is a MCMCParams object with 6 chains.

e14Tagm

## Object of class "MCMCParams"
## Method: TAGM.MCMC
## Number of chains: 6

### Data exploration and convergence diagnostics

Assessing whether or not an MCMC algorithm has converged is challenging. Assessing and diagnosing convergence is an active area of research and throughout the 1990s many approaches were proposed ^41– 44^. We provide a more detailed exploration of this issue, but readers should bare in mind that the methods provided below are diagnostics and cannot guarantee convergence. We direct readers to several important works in the literature discussing the assessment of convergence. Users that do not assess convergence and base their downstream analysis on unconverged chains are likely to obtain poor quality results.

We first assess convergence using a parallel chains approach. We find producing multiple chains is benificial not only for computational advantages but also for analysis of convergence of our chains.

## Get number of chains
nChains <- length(e14Tagm)
nChains

## [1] 6

The following code chunks set up a manual convergence diagnostic check. We make use of objects and methods in the package *coda* to perform this analysis ^45^. Our function below automatically coerces our objects into *coda* for ease of analysis. We first calculate the total number of outliers at each iteration of each chain and, if the algorithm has converged, this number should be the same (or very similar) across all 6 chains.

## Convergence diagnostic to see if we need to discard any
## iterations or entire chains: compute the number of outliers for
## each iteration for each chain
out <- mcmc_get_outliers(e14Tagm)

**Figure 6.**
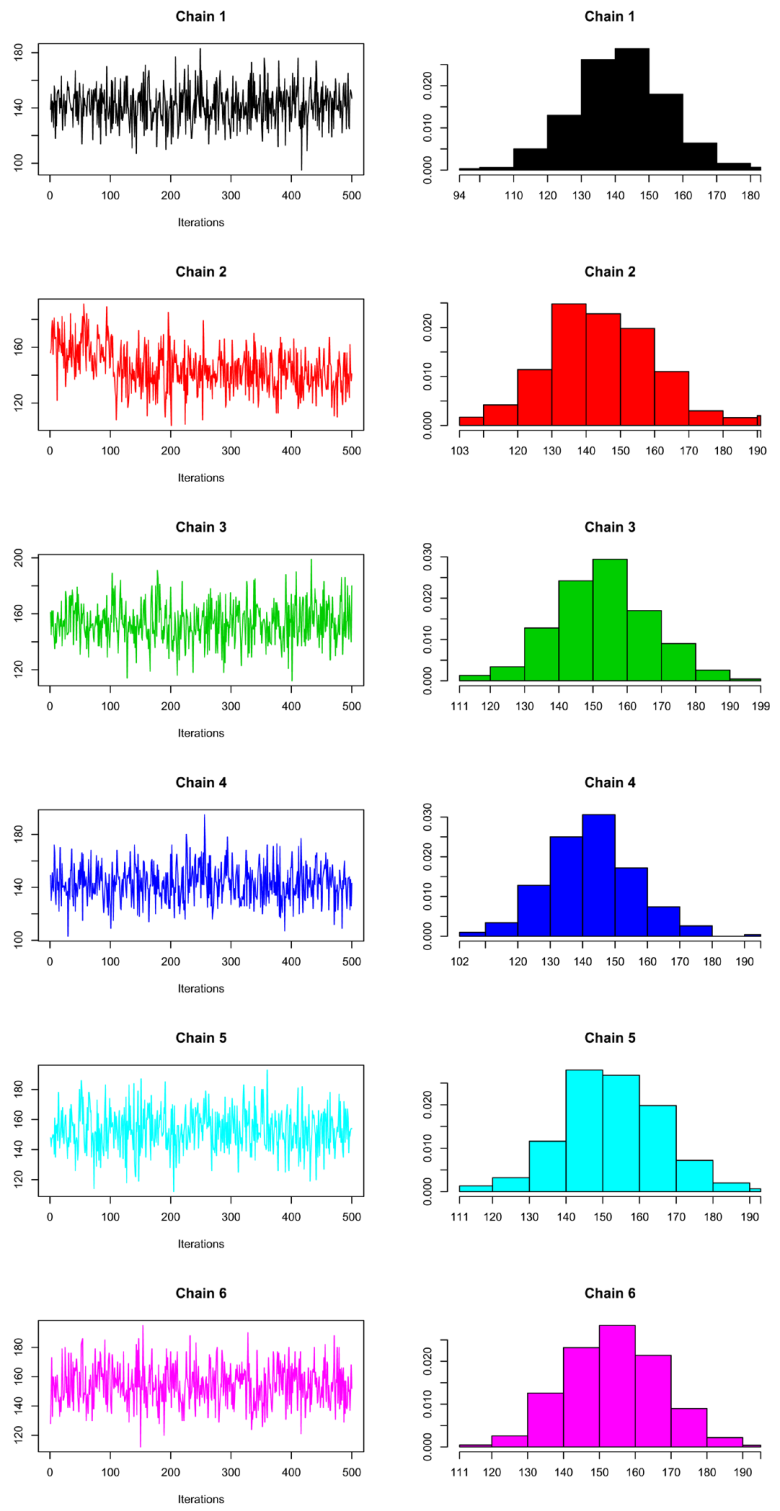
Trace (left) and density (right) of the 6 MCMC chains.

We can observe this from the trace plots and histograms for each MCMC chain ( Figure 6). Unconverged chains should be discarded from downstream analysis.

## Using coda S3 objects to produce trace plots and histograms
for (i in seq_len(nChains))
    plot(out[[i]], main = paste("Chain", i), auto.layout = FALSE, col = i)

Chains 3, 5 and 6 are centred around an average of 153, with rapid back and forth oscillations. Chain 2 should be immediately discarded, since it has a large jump in the chain with clearly skewed histogram. The other two chains oscillate differently with contrasting quantiles to the 3 chains (3, 5 and 6) that agree with one another, suggesting these chains have yet to converge. We can use the *coda* package to produce summaries of our chains. Here is the coda summary for the third chain.

## Chains average around 153 outliers
summary(out[[3]])

##
## Iterations = 1:500
## Thinning interval = 1
## Number of chains = 1
## Sample size per chain = 500
##
## 1. Empirical mean and standard deviation for each variable,
##    plus standard error of the mean:
##
##           Mean             SD       Naive SE Time-series SE
##       153.4520        14.0771         0.6295         0.6820
##
## 2. Quantiles for each variable:
##
##  2.5%   25%   50%   75% 97.5%
##   127   144   153   162   183

### Applying the Gelman diagnostic

So far, our analysis appears promising. Three of our chains are centred around an average of 153 outliers and there is no observed monotonicity in our output. However, for a more rigorous and unbiased analysis of convergence we can calculate the Gelman diagnostic using the *coda* package ^42, 44^. This statistic is often referred to as $\hat{R}$ or the potential scale reduction factor. The idea of the Gelman diagnostics is to compare the inter and intra chain variances. The ratio of these quantities should be close to one. A more detailed and in depth discussion can be found in the references. The *coda* package also reports the 95% upper confidence interval of the $\hat{R}$ statistic. In this case, our samples are approximately normally distributed (see histograms on the right in Figure 6). The *coda* package allows for transformations to improve normality of the data, and in some cases we set the transform argument to apply log transformation. Gelman and Rubin ^42^ suggest that chains with $\hat{R}$ value of less than 1.2 are likely to have converged.

gelman.diag(out, transform = FALSE)

## Potential scale reduction factors:
##
##      Point est. Upper C.I.
## [1,]       1.14       1.32

gelman.diag(out[c(1,3,4,5,6)], transform = FALSE)

## Potential scale reduction factors:
##
##      Point est. Upper C.I.
## [1,]       1.13       1.31

gelman.diag(out[c(3,5,6)], transform = FALSE)

## Potential scale reduction factors:
##
##      Point est.  Upper C.I.
## [1,]          1        1.01

In all cases, we see that the Gelman diagnostic for convergence is < 1.2. However, the upper confidence interval is 1.32 when all chains are used; 1.31 when chain 2 is removed and when chains 1, 2 and 4 are removed the upper confidence interval is 1.01 indicating that the MCMC algorithm for chains 3,5 and 6 might have converged.

We can also look at the Gelman diagnostics statistics for groups or pairs of chains. The first line below computes the Gelman diagnostic across the first three chains, whereas the second calculates the diagnostic between chain 3 and chain 5.

gelman.diag(out[1:3], transform = FALSE) # the upper C.I is 1.62

## Potential scale reduction factors:
##
##      Point est.  Upper C.I.
## [1,]       1.22        1.62

gelman.diag(out[c(3,5)], transform = TRUE) # the upper C.I is 1.01

## Potential scale reduction factors:
##
##      Point est.  Upper C.I.
## [1,]       1.01        1.01

**Figure 7.**
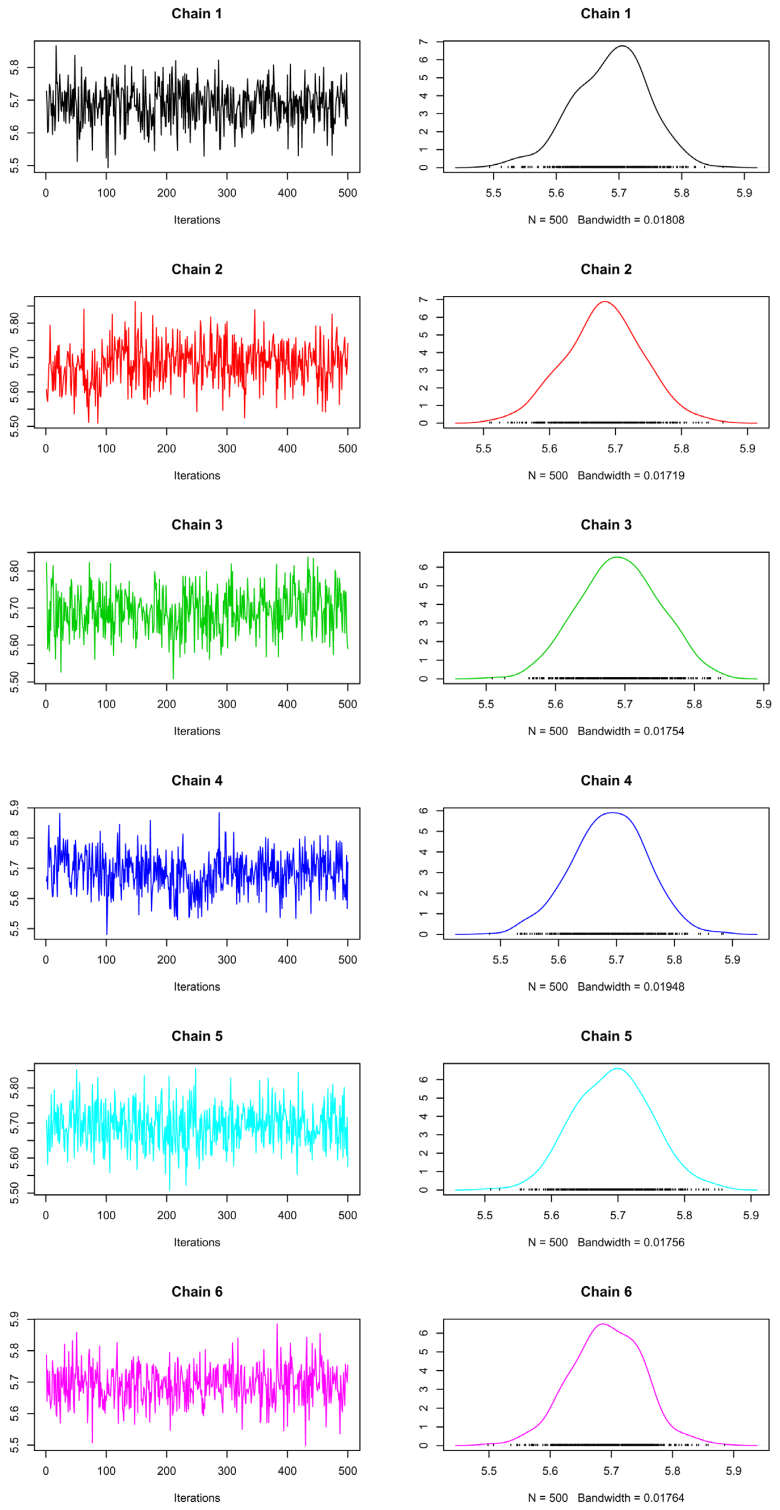
Trace (left) and density (right) of the mean component allocation of the 6 MCMC chains.

To assess another summary statistic, we can look at the mean component allocation at each iteration of the MCMC algorithm and as before we produce trace plots of this quantity ( Figure 7).

meanAlloc <- mcmc_get_meanComponent(e14Tagm)

for (i in seq_len(nChains))
     plot(meanAlloc[[i]], main = paste("Chain", i), auto.layout = FALSE, col = i)

As before we can produce summaries of the data.

summary(meanAlloc[[1]])

##
## Iterations = 1:500
## Thinning interval = 1
## Number of chains = 1
## Sample size per chain = 500
##
## 1. Empirical mean and standard deviation for each variable,
##    plus standard error of the mean:
##
##           Mean             SD      Naive  SE   Time-series SE
##       5.686713       0.059112       0.002644         0.002644
##
## 2. Quantiles for each variable:
##
##  2.5%   25%   50%   75% 97.5%
## 5.552 5.646 5.692 5.728 5.795

We can already observe that there are some slight difference between these chains which raises suspicion that some of the chains may not have converged. For example each chain appears to be centred around 5.7, but chains 2 and 4 have clear jumps in the their trace plots. For a more quantitative analysis, we again apply the Gelman diagnostics to these summaries.

gelman.diag(meanAlloc)


## Potential scale reduction factors:
##
##      Point est. Upper C.I.
## [1,]          1       1.01


The above values are close to 1 and so we there are no significant difference between the chains. As observed previously, chains 2 and 4 look quite different from the other chains and so we recalculate the diagnostic excluding these chains. The computed Gelman diagnostic below suggest that chains 3, 5 and 6 have converged and that we should discard chains 1, 2 and 4 from further analysis.

gelman.diag(meanAlloc[c(3,5,6)])


## Potential scale reduction factors:
##
##      Point est. Upper C.I.
## [1,]          1          1


For a further check, we can look at the mean outlier probability at each iteration of the MCMC algorithm and again computing the Gelman diagnostics between chains 4, 5 and 6. An $\hat{R}$ statistics of 1 is indicative of convergence, since it is less than the recommend value of 1.2.

meanoutProb <- mcmc_get_meanoutliersProb(e14Tagm)
gelman.diag(meanoutProb[c(3, 5, 6)])



## Potential scale reduction factors:
##
##      Point est. Upper C.I.
## [1,]          1       1.01


### Applying the Geweke diagnostic

Along with the Gelman diagnostic, which uses parallel chains, we can also apply a single chain analysis using the Geweke diagnostic ^41^. The Geweke diagnostic tests to see whether the mean calculated from the first 10% of iterations is significantly different from the mean calculated from the last 50% of iterations. If they are significantly different, at say a level 0.01, then this is evidence that particular chains have not converged. The following code chunk calculates the Geweke diagnostic for each chain on the summarising quantities we have previously computed.

geweke_test(out)



##           chain 1      chain 2  chain 3    chain 4    chain 5    chain 6
## z.value 0.5749775 8.816632e+00 0.470203 -0.3204500 -0.6270787 -0.7328168
## p.value 0.5653065 1.179541e-18 0.638210  0.7486272  0.5306076  0.4636702



geweke_test(meanAlloc)



##           chain 1       chain 2    chain 3    chain 4   chain 5    chain 6
## z.value 1.1952967 -3.3737051063 -1.2232102 2.48951993 0.3605882 -0.1358850
## p.value 0.2319711  0.0007416377  0.2212503 0.01279157 0.7184073  0.8919122


geweke_test(meanoutProb)


##           chain 1      chain 2   chain 3    chain 4    chain 5     chain 6
## z.value 0.1785882 1.205500e+01 0.6189637 -0.5164987 -0.2141086 -0.02379004
## p.value 0.8582611 1.825379e-33 0.5359403  0.6055062  0.8304624  0.98102008


The first test suggests chain 2 has not converged, since the p-value is less than 10 ^−10^ suggesting that the mean in the first 10% of iterations is significantly different from those in the final 50%. Moreover, the second test and third tests also suggest that chain 2 has not converged. Furthermore, for the second test chain 4 has a marginally small p-value, providing further evidence that this chain is of low quality. These convergence diagnostics are not limited to the quantities we have computed here and further diagnostics can be performed on any summary of the data.

An important question to consider is whether removing an early portion of the chain might lead to an improvement of the convergence diagnostics. This might be particularly relevant if a chain converges some iterations after our orginally specified burn-in. For example, let us take the second Geweke test above, which suggested chains 2 and 4 had not converged and see if discarding the initial 10% of the chain improves the statistic. The function below removes 50 samples, known as burn-in, from the beginning of each chain and the output shows that we now have 450 samples in each chain. In practice, as 2 chains are sufficient for good posterior estimates and convergence we could simply discard chains 2 and 4 and proceed with downstream analysis with the remaining chains.

burn_e14Tagm <-  mcmc_burn_chains(e14Tagm, 50)
chains(burn_e14Tagm)

burn_e14Tagm <-  mcmc_burn_chains(e14Tagm, 50)
chains(burn_e14Tagm)


## Object of class "MCMCChains"
##  Number of chains: 6


chains(burn_e14Tagm)[[4]]


## Object of class "MCMCChain"
##  Number of components: 10
##  Number of proteins: 1663
##  Number of iterations: 450

The following function recomputes the number of outliers in each chain at each iteration of each Markov-chain.

out2 <- mcmc_get_outliers(burn_e14Tagm)

The code chuck below computes the Geweke diagnostic for this new truncated chain and demonstrates that chain 4 has an improved Geweke diagnostic, whilst chain 2 does not. Thus, in practice, it maybe useful to remove iterations from the beginning of the chain. However, as chain 4 did not pass the Gelman diagnostics we still discard it from downstream analysis.

geweke_test(out2)



##            chain 1      chain 2    chain 3   chain 4   chain 5   chain 6
## z.value -0.1455345 6.379618e+00 -1.6392215 0.3836940 0.1241201 0.6654703
## p.value  0.8842889 1.775298e-10  0.1011671 0.7012053 0.9012202 0.5057497




### Processing converged chains

Having made an assessment of convergence, we decide to discard chains 1,2 and 4 from any further analysis. The code chunk below removes these chains and creates a new object to store the converged chains.

removeChain <- c(1, 2, 4) # The chains to be removed
e14Tagm_converged <- e14Tagm[-removeChain] # Create new object



The MCMCParams object can be large and therefore if we have a large number of samples we may want to subsample our chain, known as *thinning*, to reduce the number of samples. Thinning also has another purpose. We may desire independent samples from our posterior distribution but the MCMC algorithm produces autocorrelated samples. Thinning can be applied to reduce the auto-correlation between samples. The code chuck below, which is not evaluated, demonstrates retaining every 5 *^th^* iteration. Recall that we thinned by 20 when we first ran the MCMC algorithm.

e14Tagm_converged_thinned <- mcmc_thin_chains(e14Tagm_converged, freq = 5)

We initially ran 6 chains and, after having made an assessment of convergence, we decided to discard 3 of the chains. We desire to make inference using samples from all 3 chains, since this leads to better posterior estimates. In their current class structure all the chains are stored separately, so the following function pools all sample for all chains together to make a single longer chain with all samplers. Pooling a mixture of converged and unconverged chains is likely to lead to poor quality results so should be done with care.

e14Tagm_converged_pooled <- mcmc_pool_chains(e14Tagm_converged)
e14Tagm_converged_pooled

## Object of class "MCMCParams"
## Method: TAGM.MCMC
## Number of chains: 1

e14Tagm_converged_pooled[[1]]

## Object of class "MCMCChain"
##  Number of components: 10
##  Number of proteins: 1663
##  Number of iterations: 1500

To populate the summary slot of the converged and pooled chain, we can use the tagmMcmcProcess function. As we can see from the object below a summary is now available. The information now available in the summary slot was detailed in the previous section. We note that if there is more than 1 chain in the MCMCParams object then the chains are automatically pooled to compute the summaries.

e14Tagm_converged_pooled <- tagmMcmcProcess(e14Tagm_converged_pooled)
e14Tagm_converged_pooled

## Object of class "MCMCParams"
## Method: TAGM.MCMC
## Number of chains: 1
## Summary available

To create new feature columns in the MSnSet and append the summary information, we apply the tagmPredict function. The probJoint argument indicates whether or not to add probabilistic information for all organelles for all proteins, rather than just the information for the most probable organelle. The outlier probabilities are also returned by default, but users can change this using the probOutlier argument.

E14TG2aR <- tagmPredict(object = E14TG2aR,
                           params = e14Tagm_converged_pooled,
                           probJoint = TRUE)
head(fData(E14TG2aR))

##        Uniprot.ID UniprotName
## Q62261     Q62261 SPTB2_MOUSE
## Q9JHU4     Q9JHU4 DYHC1_MOUSE
## Q9QXS1     Q9QXS1  PLEC_MOUSE
##                                     Protein.Description Peptides PSMs
## Q62261 Spectrin beta chain, brain 1 (multiple isoforms)       42   42
## Q9JHU4               Cytoplasmic dynein 1 heavy chain 1       33   33
## Q9QXS1                       Isoform PLEC-1I of Plectin       33   33
##        GOannotation markers.orig markers   tagm.map.allocation
## Q62261      PLM-SKE      unknown unknown Endoplasmic reticulum
## Q9JHU4          SKE      unknown unknown   Nucleus - Chromatin
## Q9QXS1      unknown      unknown unknown       Plasma membrane
##        tagm.map.probability tagm.map.outlier  tagm.mcmc.allocation
## Q62261         8.165817e-09     0.9999999857 Endoplasmic reticulum
## Q9JHU4         9.996798e-01     0.0003202255   Nucleus - Chromatin
## Q9QXS1         1.250898e-06     0.9999987491            Proteasome
##        tagm.mcmc.probability tagm.mcmc.probability.lowerquantile
## Q62261             0.5765793                        0.0020296117
## Q9JHU4             0.9738206                        0.7594516090
## Q9QXS1             0.4957129                        0.0002886457
##        tagm.mcmc.probability.upperquantile tagm.mcmc.mean.shannon
## Q62261                           0.9992504            0.201623229
## Q9JHU4                           0.9998822            0.081450206
## Q9QXS1                           0.9947100            0.447665536
##        tagm.mcmc.outlier tagm.mcmc.joint.40S Ribosome
## Q62261      2.547793e-01                 4.401228e-10
## Q9JHU4      3.335134e-05                 1.936225e-18
## Q9QXS1      6.423799e-01                 2.213861e-07
##        tagm.mcmc.joint.60S Ribosome tagm.mcmc.joint.Cytosol
## Q62261                 2.778620e-07            2.650861e-12
## Q9JHU4                 1.645727e-21            1.887645e-17
## Q9QXS1                 1.495170e-01            9.062280e-09
##        tagm.mcmc.joint.Endoplasmic reticulum tagm.mcmc.joint.Lysosome
## Q62261                          5.765793e-01             1.108757e-11
## Q9JHU4                          1.548053e-17             5.577415e-24
## Q9QXS1                          1.768681e-04             1.150706e-04
##        tagm.mcmc.joint.Mitochondrion tagm.mcmc.joint.Nucleus - Chromatin
## Q62261                  5.020528e-08                        4.231731e-01
## Q9JHU4                  2.835919e-22                        9.738206e-01
## Q9QXS1                  5.832273e-19                        7.920397e-03
##        tagm.mcmc.joint.Nucleus - Nucleolus tagm.mcmc.joint.Plasma membrane
## Q62261                        1.279255e-05                    1.914808e-11
## Q9JHU4                        2.617943e-02                    3.514851e-29
## Q9QXS1                        1.130580e-05                    3.465462e-01
##        tagm.mcmc.joint.Proteasome
## Q62261               2.345204e-04
## Q9JHU4               7.841425e-11
## Q9QXS1               4.957129e-01
##  [ reached getOption("max.print") -- omitted 2 rows ]
##  [ reached ’max’ / getOption("max.print") -- omitted 1 rows ]


### Aside: *Priors*

Bayesian analysis requires users to specify prior information about the parameters. This may appear to be a challenging task; however, good default options are often possible. Should expert information be available for any of these priors then the users should provide this, otherwise we have found that the default choices work well in practice. The priors also provide regularisation and shrinkage to avoid overfitting. Given enough data the likelihood overwhelms the prior and the influence of the prior is weak.

We place a normal inverse-Wishart prior on the parameters of the mutivariate normal mixture components. The normal inverse-Wishart prior has 4 hyperparameters that must be specified. These are: the prior mean mu0 expressing the prior location of each organelle; a prior shrinkage lambda0, which is a scalar expressing uncertainty in the prior mean; the prior degrees of freedom nu0; and a scale prior S0 on the covariance. Together, nu0 and S0 specify the prior variability on organelle covariances. The same prior distribution is assumed for the parameters of all mutivariate normal mixture components.

The default options for these are based on the choice recommended by ^46^. The prior mean mu0 is set to be the mean of the data. lambda0 is set to be 0.01 meaning some uncertainty in the covariance is propagated to the mean, increasing lambda0 increases shrinkage towards the prior. nu0 is set to the number of feature variables plus 2, which is the smallest integer value that ensures a finite covariance matrix. The prior scale matrix *S*0 is set to

$$S_{0}=\frac{\mathrm{diag}\text{(}\frac{\text{1}}{n}\sum {\text{(}X-\bar{X}\text{)}}^{\text{2}}\text{)}}{K^{1/\text{D}}},\;(1)$$

and represents a diffuse prior on the covariance. Another good choice which is often used is a constant multiple of the identity matrix. The prior for the Dirichlet distribution concentration parameters beta0 is set to 1 for each organelle. Another reasonable choice would be the non-informative Jeffery’s prior for the Dirichlet hyperparameter, which sets beta0 to 0.5 for each organelle. The prior weight for the outlier detection class is a *ℬ* ( *u*, *v*) distribution. The default for *u* = 2 and the default for *v* = 10. This represents the reasonable belief that $\frac{u}{u+v}=\frac{1}{6}$ proteins *a priori* might be an outlier and we believe is unlikely that more than 50% of proteins are outliers. Decreasing the value of *v*, represents more uncertainty about the number of protein that are outliers.

### Analysis, visualisation and interpretation of results

**Figure 8.**
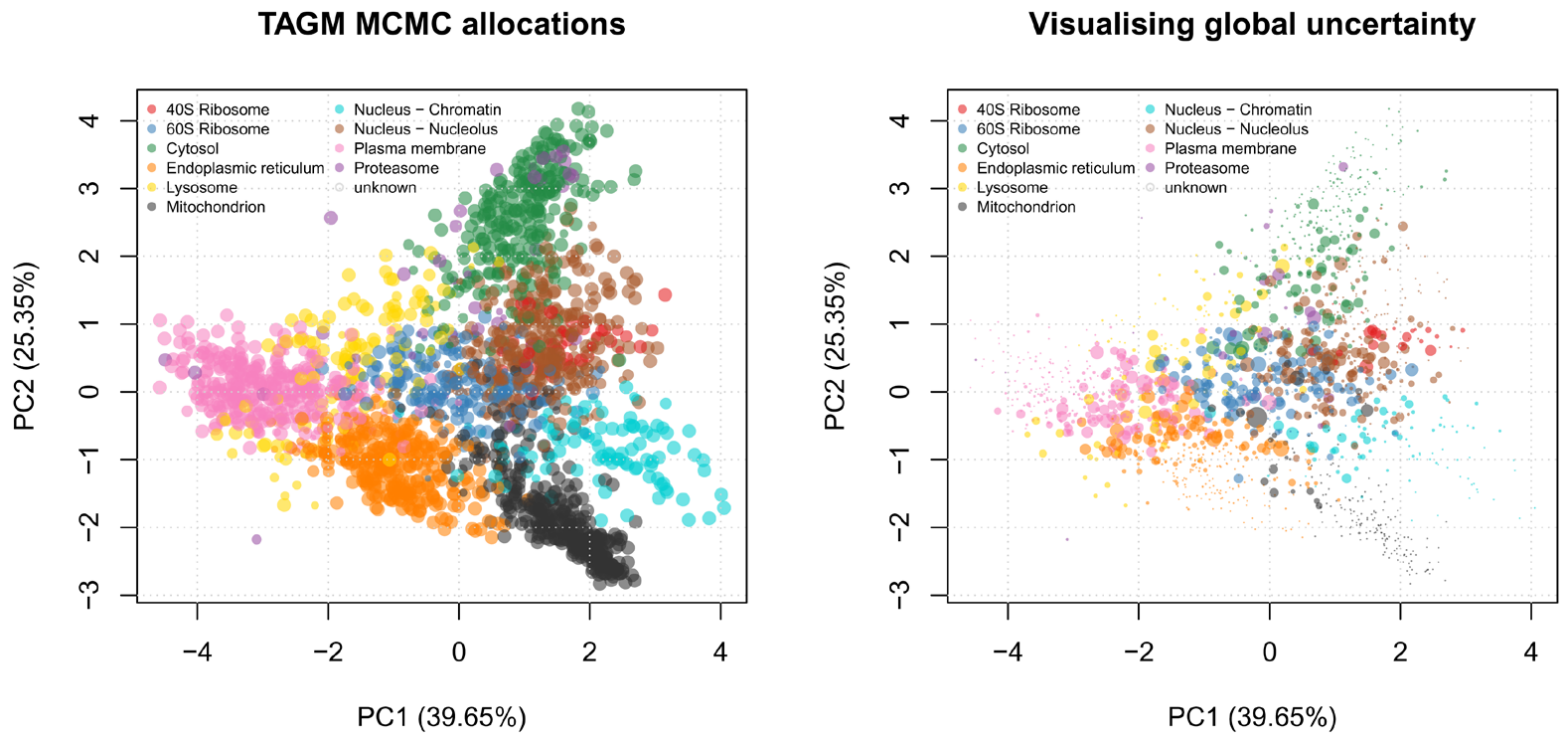
TAGM MCMC allocations. On the left, point size have been scaled based on allocation probabilities. On the right, the point size have been scaled based on the global uncertainty using the mean Shannon entropy.

Now that we have a single pooled chain of samples from a converged MCMC algorithm, we can begin to analyse the results. Preliminary analysis includes visualising the allocated organelle and localisation probability of each protein to its most probable organelle, as shown on Figure 8.

par(mfrow = c(1, 2))
plot2D(E14TG2aR, fcol = "tagm.mcmc.allocation",
        cex = fData(E14TG2aR)$tagm.mcmc.probability,
        main = "TAGM MCMC allocations")
addLegend(E14TG2aR, fcol = "markers",
           where = "topleft", ncol = 2, cex = 0.6)

plot2D(E14TG2aR, fcol = "tagm.mcmc.allocation",
        cex = fData(E14TG2aR)$tagm.mcmc.mean.shannon,
        main = "Visualising global uncertainty")
addLegend(E14TG2aR, fcol = "markers",
           where = "topleft", ncol = 2, cex = 0.6)

We can visualise other summaries of the data including a Monte-Carlo averaged Shannon entropy, as shown in Figure 8 on the right. This is a measure of uncertainty and proteins with greater Shannon entropy have more uncertainty in their localisation. We observe global patterns of uncertainty, particularly in areas where organelle boundaries overlap. There are also regions of low uncertainty indicating little doubt about the localisation of these proteins.

**Figure 9.**
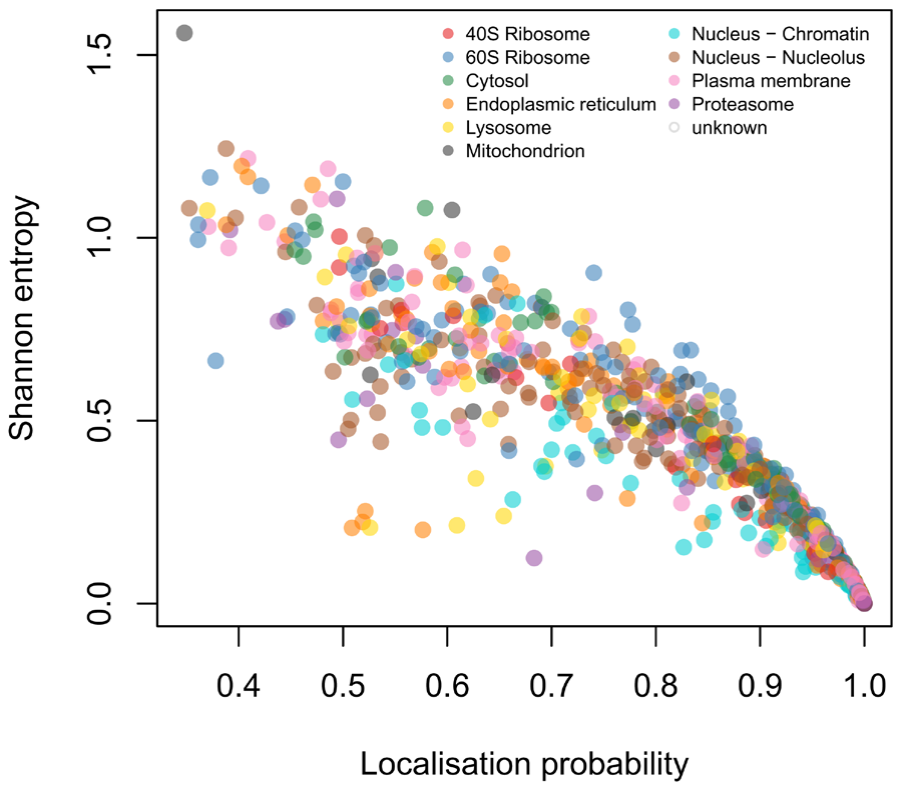
Shannon entropy and localisation probability.

We are also interested in the relationship between localisation probability to the most probable class and the Shannon entropy ( Figure 9). Even though the two quantities are evidently correlated there is still considerable spread. Thus it is important to base inference not only on localisation probability but also a measure of uncertainty, for example the Shannon entropy. Proteins with low Shannon entropy have low uncertainty in their localisation, whilst those with higher Shannon entropy have uncertain localisation. Since multi-localised protein have uncertain localisation to a single subcellular niche, exploring the Shannon can aid in identifying multi-localised proteins.

cls <- getStockcol()[as.factor(fData(E14TG2aR)$tagm.mcmc.allocation)]
plot(fData(E14TG2aR)$tagm.mcmc.probability,
     fData(E14TG2aR)$tagm.mcmc.mean.shannon,
     col = cls, pch = 19,
     xlab = "Localisation probability",
     ylab = "Shannon entropy")
addLegend(E14TG2aR, fcol = "markers",
           where = "topright", ncol = 2, cex = 0.6)

**Figure 10.**
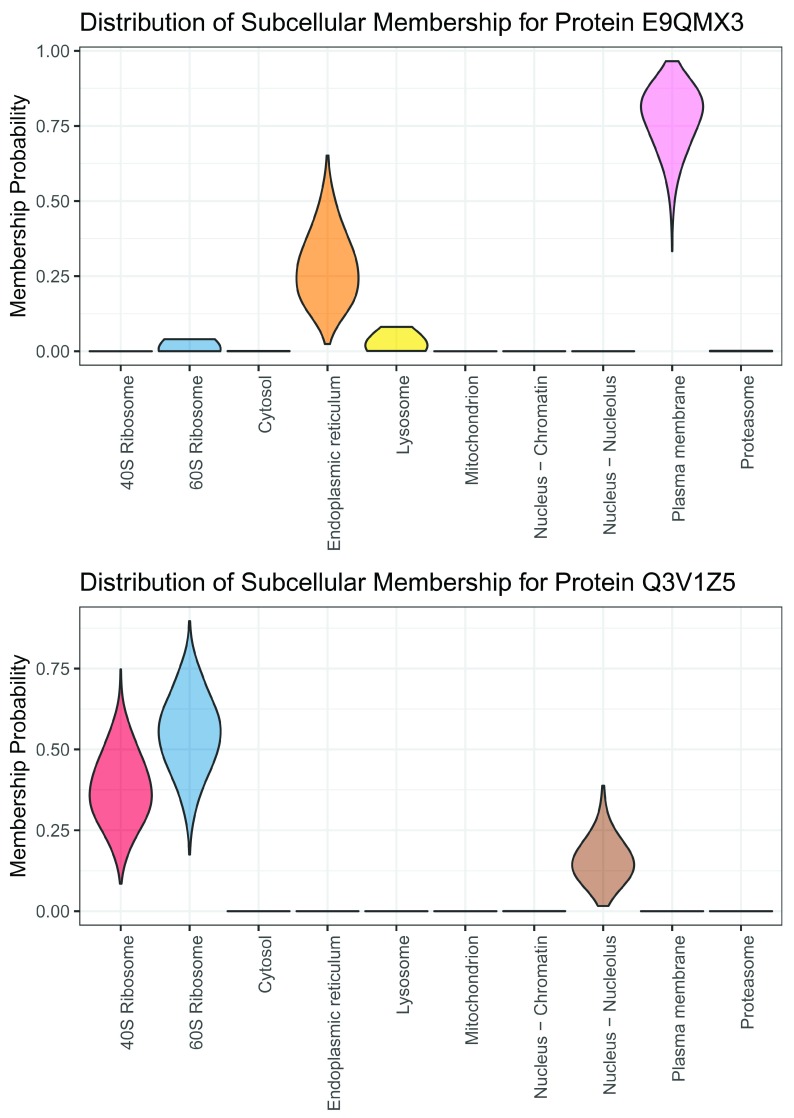
Full posterior distribution of localisation probabilities for individual proteins.

Aside from global visualisation of the data, we can also interrogate each individual protein. As illustrated on Figure 10, we can obtain the full posterior distribution of localisation probabilities for each protein from the e14Tagm_converged_pooled object. We can use the plot generic on the MCMCParams object to obtain a violin plot of the localisation distribution. Simply providing the name of the protein in the second argument produces the plot for that protein. The solute carrier transporter protein E9QMX3, also referred to as Slc15a1, is most probably localised to plasma membrane in line with its role as a transmembrane transporter but also shows some uncertainty, potentially also localising to other comparments. The first violin plot visualises this uncertainty. The protein Q3V1Z5 is a supposed constitute of the 40S ribosome and has poor UniProt annotation with evidence only at the transcript level. From the plot below is is clear that Q3V1Z5 is a ribosomal associated protein, but it previous localisation has only been computational inferred and here we provide experimental evidence of a ribosomal annotation. Thus, quantifying uncertainty recovers important additional annotations.

plot(e14Tagm_converged_pooled, "E9QMX3")
plot(e14Tagm_converged_pooled, "Q3V1Z5")

## Discussion

The Bayesian analysis of biological data is of clear interest to many because of its ability to provide richer information about the experimental results. A fully Bayesian analysis differs from other machine learning approaches, since it can quantify the uncertainty in our inferences. Furthermore, we use a generative model to explicitly describe the data, which makes inferences more interpretable compared to the less interpretable outputs of black-box classifiers such as, for example, support vector machines (SVM).

Bayesian analysis is often characterised by its provision of a (posterior) probability distribution over the biological parameters of interest, as opposed to single point estimate of these parameters. In the case that is presented in this workflow, a Bayesian analysis “computes” a posterior probability distribution over the protein localisation probabilities. These probability distributions can then be rigorously interrogated for greater biological insight; in addition, it may allow us to ask additional questions about the data, such as whether a protein might be multi-localised.

Despite the wealth of information a Bayesian analysis can provide, the uptake amongst cell biologists is still low. This is because a Bayesian analysis presents a new set of challenges and little practical guidance exists regarding how to address these challenges. Bayesian analyses often rely on computatinally intensive approaches such as Markov-chain Monte-Carlo (MCMC) and a practical understanding of these algorithms and the interpretation of their output is a key barrier to their use. A Bayesian analysis usually consists of three broad steps:

(1) Data pre-processing and algorithmic implementation, (2) assessing algorithmic convergence and (3) summarising and visualising the results. This workflow provides a set of tools to simplify these steps and provides step-by-step guidance in the context of the analysis of spatial proteomics data.

We have provided a workflow for the Bayesian analysis of spatial proteomics using the pRoloc and MSnbase software. We have demonstrated, in a step-by-step fashion, the challenges and advantages associated with taking a Bayesian approach to data analysis. We hope this workflow will help spatial proteomics practitioners to apply our methods and will motivate others to create detailed documentation for the Bayesian analysis of biological data.

## Session information

Below, we provide a summary of all packages and versions used to generate this document.

sessionInfo()

## R version 3.5.2 Patched (2019-01-24 r76018)
## Platform: x86_64-pc-linux-gnu (64-bit)
## Running under: Manjaro Linux
##
## Matrix products: default
## BLAS: /usr/lib/libblas.so.3.8.0
## LAPACK: /usr/lib/liblapack.so.3.8.0
##
## locale:
##  [1] LC_CTYPE=en_US.UTF-8       LC_NUMERIC=C
##  [3] LC_TIME=en_US.UTF-8        LC_COLLATE=en_US.UTF-8
##  [5] LC_MONETARY=en_US.UTF-8    LC_MESSAGES=en_US.UTF-8
##  [7] LC_PAPER=en_US.UTF-8       LC_NAME=C
##  [9] LC_ADDRESS=C               LC_TELEPHONE=C
## [11] LC_MEASUREMENT=en_US.UTF-8 LC_IDENTIFICATION=C
##
## attached base packages:
## [1] stats4    parallel  stats     graphics  grDevices utils     datasets
## [8] methods   base
##
## other attached packages:
##  [1] patchwork_0.0.1      pRolocdata_1.21.1    pRoloc_1.23.2
##  [4] coda_0.19-2          mixtools_1.1.0       BiocParallel_1.16.6
##  [7] MLInterfaces_1.62.0  cluster_2.0.7-1      annotate_1.60.1
## [10] XML_3.98-1.19        AnnotationDbi_1.44.0 IRanges_2.16.0
## [13] MSnbase_2.9.3        ProtGenerics_1.14.0  S4Vectors_0.20.1
## [16] mzR_2.17.2           Rcpp_1.0.1           Biobase_2.42.0
## [19] BiocGenerics_0.28.0
##
## loaded via a namespace (and not attached):
##   [1] tidyselect_0.2.5        RSQLite_2.1.1
##   [3] htmlwidgets_1.3         grid_3.5.2
##   [5] trimcluster_0.1-2.1     lpSolve_5.6.13
##   [7] rda_1.0.2-2.1           devtools_2.0.1
##   [9] munsell_0.5.0           codetools_0.2-16
##  [11] preprocessCore_1.44.0   withr_2.1.2
##  [13] colorspace_1.4-1        knitr_1.22
##  [15] rstudioapi_0.10         robustbase_0.93-4
##  [17] mzID_1.20.1             labeling_0.3
##  [19] git2r_0.25.2            hwriter_1.3.2
##  [21] bit64_0.9-7             ggvis_0.4.4
##  [23] rprojroot_1.3-2         generics_0.0.2
##  [25] ipred_0.9-8             xfun_0.5
##  [27] randomForest_4.6-14     diptest_0.75-7
##  [29] R6_2.4.0                doParallel_1.0.14
##  [31] flexmix_2.3-15          bitops_1.0-6
##  [33] assertthat_0.2.0        promises_1.0.1
##  [35] scales_1.0.0            nnet_7.3-12
##  [37] gtable_0.2.0            affy_1.60.0
##  [39] processx_3.3.0          timeDate_3043.102
##  [41] rlang_0.3.1             genefilter_1.64.0
##  [43] splines_3.5.2           lazyeval_0.2.2
##  [45] ModelMetrics_1.2.2      impute_1.56.0
##  [47] hexbin_1.27.2           BiocManager_1.30.4
##  [49] yaml_2.2.0              reshape2_1.4.3
##  [51] threejs_0.3.1           crosstalk_1.0.0
##  [53] backports_1.1.3         httpuv_1.5.0
##  [55] caret_6.0-81            tools_3.5.2
##  [57] lava_1.6.5              usethis_1.4.0
##  [59] bookdown_0.9            ggplot2_3.1.0
##  [61] affyio_1.52.0           RColorBrewer_1.1-2
##  [63] proxy_0.4-23            sessioninfo_1.1.1
##  [65] plyr_1.8.4              base64enc_0.1-3
##  [67] progress_1.2.0          zlibbioc_1.28.0
##  [69] purrr_0.3.2             RCurl_1.95-4.12
##  [71] ps_1.3.0                prettyunits_1.0.2
##  [73] rpart_4.1-13            viridis_0.5.1
##  [75] sampling_2.8            sfsmisc_1.1-3
##  [77] LaplacesDemon_16.1.1    fs_1.2.7
##  [79] magrittr_1.5            data.table_1.12.0
##  [81] pcaMethods_1.74.0       mvtnorm_1.0-10
##  [83] whisker_0.3-2           pkgload_1.0.2
##  [85] hms_0.4.2               mime_0.6
##  [87] evaluate_0.13           xtable_1.8-3
##  [89] mclust_5.4.3            gridExtra_2.3
##  [91] testthat_2.0.1          compiler_3.5.2
##  [93] biomaRt_2.38.0          tibble_2.1.1
##  [95] ncdf4_1.16.1            crayon_1.3.4
##  [97] htmltools_0.3.6         segmented_0.5-3.0
##  [99] later_0.8.0             BiocWorkflowTools_1.8.0
##  [ reached getOption("max.print") -- omitted 49 entries ]


The source of this document, including the code necessary to reproduce the analyses and figures is available in a public manuscript repository on GitHub ^47^.

## Data availability

The data used in this workflow was first published in Breckels **et al*.* (2016) ^36^ and is available in the pRolocdata package.

## Software availability

Computational workflow for this study available from: https://github.com/ococrook/TAGMworkflow ^47^

Archived source code at time of publication: https://doi.org/10.5281/zenodo.2593712 ^33^

License: CC BY 4.0

## Footnotes

^1^ https://drive.google.com/open?id=1zozntDhE6YZ-q8wjtQ-lxZ66EEszOGYi
