# Supplementary material for: A Bioconductor workflow for the Bayesian analysis of spatial proteomics
Source: F1000Res. 2019 Apr 11;8:446. [Version 1] doi: 10.12688/f1000research.18636.1 (PMC6509962; doi:10.12688/f1000research.18636.1)
Supplement: Peer review history

# Peer review: Reviewer response for version 1

**DOI:** 10.5256/f1000research.20403.r47080

**Published:** 2019-05-09

**Authors:** Angus I Lamond, Alejandro Brenes Murillo

**Affiliations:** 1 Centre for Gene Regulation and Expression, School of Life Sciences, University of Dundee, Dundee, UK; 2 University of Dundee, Dundee, UK

The subcellular distribution of the proteome is a very important determinant of cell function and therefore the ability to analyse subcellular protein localisation is vital for studying cell biology and regulation. Consequently, technical developments that facilitate the analysis of how the proteome is distributed between cell compartments are of great value to both the proteomics and molecular cell biology research communities. Bioconductor is also a fantastic source of tools that can be used to analyse ‘omics’ datasets, hence further additions to this toolbox are always welcome and of value. Here the authors provide a detailed workflow in Bioconductor that enables the convenient Bayesian analysis of spatial proteomics data. The explanations of the models provided by the authors are user friendly and allow also non-bioinformaticians to understand clearly the mechanics of the different methods. The suggestions provided relating to default parameters for the models are also very welcome. Overall, I view this as a timely and very useful contribution for the community that will be well received and of genuine value. I note below a few specific points that the authors should address.

 Specific Points:

- I was initially unable to load either pRoloc or pRolocdata on R and therefore could not follow any of the examples provided. I had to upgrade to a new version of R to resolve this issue. The authors should specify the R version compatibility required.
- Line 1: Remove one ‘the’
- 3rd page 5 paragraph, second sentence is disconnected.
- Example dataset uses iTRAQ, which means the data will have false positives all across
- The authors should explain/justify their assertion that their Method is independent of the isotope labelling method because TMT and iTRAQ are expected to provide different challenges?
- It would be helpful for the Gelman diagnostic to suggest minimum a number of chains for MCMC
  - Suggestion for an ideal number of chains for a medium range desktop computer would be useful too
- “Trace for Chain 2 & 4 have clear jumps” This is for figure 7
  - This sounds imprecise; hard to spot and replicate. Also, I do not see the “clear jumps” the authors refer to – can this be clarified?
- Suggesting default priors for the Bayesian analysis is very useful. However, it would be good to show the visual consequences of changing S0 from beta0 of 1 to beta0 of 0.5
- The suggestion by the authors that it is likely that 16.7% of proteins are outliers needs to be justified.
- The use of Shannon entropy to focus on proteins with multiple localisations is an interesting approach that could be discussed further.

We have read this submission. We believe that we have an appropriate level of expertise to confirm that it is of an acceptable scientific standard.

# Peer review: Reviewer response for version 1

**DOI:** 10.5256/f1000research.20403.r47076

**Published:** 2019-05-01

**Authors:** Susan P. Holmes

**Affiliations:** 1 Department of Statistics, Stanford University, Stanford, CA, USA

This paper provides an important advance in the study of spatial proteomics. Through the use of Monte Carlo sampling the authors are able to provide evaluations of uncertainty by using the T-augmented Gaussian mixture model proposed by Crook et al. 

 The authors have been careful to provide all the code and the relevant data are included in the package 'pRolocdata'.

 Although the focus is on the spatial localisation of proteins, the authors only study the probabilities of correct localizations and do not show a spatial plot of the probabilities so one does not see clearly how the probabilities vary spatially.

**First Issue: PCA plots**

 a) Ratios:

 There are a few corrections needed to the figures that show PCA plots. It is probably not worth showing the percentages of variance up to two decimal digits, however it is important to respect the axes relative scale and units. For instance, since the ratio of variances in Figure 1 is 40:25 the plot should be rectangular, this is more egregious in Figures 2, especially the right side panel. I suggest making the figures on top of each other so they are not narrow (For a reference see Chapter 7 of the book ^1^ that explains why usually PCA plots should be rectangular and not square).

 b) Construction of ellipses on the PCA plots in Figure 2 is very confusing. These cannot be the posterior distributions and their construction presupposes there is a relevant multivariate normal distribution which I do not think is justified here.

**Second Issue: Bayesian computations**

 a) Priors

 In the "Aside" subsection on priors, I suggest exploring the possibility of using prior data to construct a prior as in practical situations this is often how pragmatic Bayesians think about building priors. I think it is worth supporting the statement that the posterior is overwhelmed by the data with a small example. This could be done following the type of process recommended by Betancourt who gives very good examples of the effect of priors through the use of *posterior predictive distributions (see here *https://betanalpha.github.io/assets/case_studies/principled_bayesian_workflow.html#24_model_adequacy *).*

 b) Convergence diagnostics

 The use of trace plots and Gelman and Rubin's Rhat can be quite confusing and can only show non-convergence, the current version puts too much emphasis on a few short runs. The authors have shown some chains that have not converged but do not mention that the contemporary literature is much more careful of how small the Rhat should be, see the preprint by Vats,D and Knudson, C 2018 ^2^ and the discussions by Dan Simpson and Andrew Gelman for instance (https://statmodeling.stat.columbia.edu/2019/03/19/maybe-its-time-to-let-the-old-ways-die-or-we-broke-r-hat-so-now-we-have-to-fix-it/), the preprint is on the arXiv ^3^.

**Third Issue:**

 Visualization of results: Figure 8 does not show the uncertainty in an intuitive way. It is very hard to differentiate the size of the dots on the left hand plot and the dots give the impression of a circular posterior distribution which is incorrect. The reference Ren et al, 2017 ^4^ contains several examples of posterior confidence contours that communicate uncertainty in a more precise way which enables the comparisons of uncertainty across different locations in the PCA. It would also be illuminating if the authors could make a plot with uncertainty contours as a function of the underlying spatial coordinates.

I have read this submission. I believe that I have an appropriate level of expertise to confirm that it is of an acceptable scientific standard, however I have significant reservations, as outlined above.

## References

[1] HolmesSWolfgangH: Modern Statistics for Modern Biology. *Cambridge University Press*.2018;

[2] VatsDKnudsonC: Revisiting the Gelman-Rubin Diagnostic. *arXiv*.2018;

[3] VehtariAGelmanASimpsonDCarpenterBBürknerP: Rank-normalization, folding, and localization: An improved Rˆ for assessing convergence of MCMC. *arXiv*.2019;

[4] RenBBacalladoSFavaroSHolmesSTrippaL: Bayesian Nonparametric Ordination for the Analysis of Microbial Communities. *J Am Stat Assoc*.2017;112(520) : 10.1080/01621459.2017.1288631 1430-1442 10.1080/01621459.2017.1288631 29430070PMC5804367

# Peer review: Reviewer response for version 1

**DOI:** 10.5256/f1000research.20403.r47079

**Published:** 2019-04-30

**Authors:** Pierre M. Jean Beltran

**Affiliations:** 1 Proteomics Platform, Broad Institute, Cambridge, USA

The spatial proteomics field has seen increased popularity over the past few years through development of experimental, statistical, and computational methodologies. In this Method Article, Crook OM and colleagues present a bioinformatics workflow for the analysis of spatial proteomics data using a set of Bayesian analysis tools. This work is a useful guide for biologists that wish to properly apply and diagnose a Markov-chain Monte-Carlo (MCMC) inference procedure using the pRoloc package.

 The article is timely and relevant given the increasing number of biologists acquiring programming skills yet lacking extensive expertise in this area of statistics and modeling. In particular, the authors did a good job explaining basic terminology and rationale for the diagnostics necessary during MCMC inference. The methodology is clearly explained, all the code is provided, and the packages and datasets are available through Bioconductor, making them easily accessible and reproducible.

 However, I believe this article lacks in background information and details for those readers that are not experts in the spatial proteomics field. In addition, the value of applying the more resource-intensive MCMC method compared to the expectation-maximisation (EM) algorithm is not clear from the data presented. I recommend this article for indexing given that the following issues are resolved.

**Major comments**

1. Currently, there is no direct comparison of the results provided by the EM and MCMC algorithms. Given that MCMC is computationally intensive and requires additional diagnostics, the reader needs to be convinced that there is a tangible benefit from using the MCMC approach.
2. A discussion of scenarios in which the EM algorithm would be preferable over MCMC would be useful for the reader. For example, can MCMC fail to converge? If so, what kind of interpretation can be obtained by looking at the point-estimates from the EM algorithm instead?
3. Additional background information and references would be useful, as this article seems to assume that the reader is familiar with spatial proteomics data sets. For example, the author can explain the organelle MS-proteomics data sets, the use of organelle markers to train the model, and the prediction of unannotated proteins from the model fitted on the organelle markers. In addition, I would suggest pointing the reader to the review by Gatto L et al. 2014 ^1^ in MCP, or another similar review as a primer.
4. Page 20 – The authors claim that lower Shannon entropy is an indication of low uncertainty in localization and therefore can aid in identifying multi-localized proteins. This claim should be backed up by some examples or evidence from the literature that agree with the data presented.

**Minor comments**

1. Figure 1 – Additional details should be provided in the figure legend. Is this plot only showing organelle markers?
2. A short description of the outlier component and how the analyst can interpret this component would be particularly helpful.
3. Page 15 – It is not clear why the Gelman diagnostic performed on mean allocation of all chains helps discriminate chains 1, 2, and 4. The upper C.I. is already near to 1 (1.01) for this example.
4. I observed several small writing mistakes; the article should be proof-read before publication. Some examples are indicated below.
5. Page 15 – sentence ending with “… Gelman diagnostics between chains 4, 5, 6”. However, the code shows that the diagnostic was performed on chains 3, 5, 6.
6. Page 21 – “From the plot below is is…” should read “it is”.

I have read this submission. I believe that I have an appropriate level of expertise to confirm that it is of an acceptable scientific standard, however I have significant reservations, as outlined above.

## References

[1] GattoLBreckelsLMBurgerTNightingaleDJGroenAJCampbellCNikolovskiNMulveyCMChristoforouAFerroMLilleyKS: A foundation for reliable spatial proteomics data analysis. *Mol Cell Proteomics*.2014;13(8) : 10.1074/mcp.M113.036350 1937-52 10.1074/mcp.M113.036350 24846987PMC4125728
